# Prognostic Significance of the Strong Ion Gap in Patients in Medical and Surgical Intensive Care Units

**DOI:** 10.7759/cureus.47964

**Published:** 2023-10-30

**Authors:** Halil Ibrahim Altun, Gozde Altun, Omer Faruk Altas, Gulcin Aran

**Affiliations:** 1 Anesthesiology and Reanimation, Kanuni Sultan Suleyman Training and Research Hospital, Istanbul, TUR; 2 Anesthesiology and Reanimation, Institute of Cardiology, Istanbul University-Cerrahpasa, Istanbul, TUR; 3 Anesthesiology and Reanimation, Bakırcay University Cigli Training and Research Hospital, Izmir, TUR; 4 Anesthesiology and Reanimation, Katip Celebi University Ataturk Training and Research Hospital, Izmir, TUR

**Keywords:** mortality, apache 2 score, acid-base imbalance, stewart approach, intensive care unit admission

## Abstract

Background

This study aimed to analyze acid-base imbalance by assessing the arterial blood gas (ABG) samples of the medical and surgical intensive care unit (ICU) patients by the Stewart approach and demonstrate the advantages of this method in delineating the acid-base status in cases where Henderson-Hasselbalch, anion gap, and base excess cannot optimally depict the imbalance and create recognition in the clinicians in this regard.

Methodology

Adult (i.e., age > 18 years) patients admitted to the ICU of our institution during a one-year study period were included in this study. The patients were divided into two groups based on the indication of admission to the ICU as *medical* or *surgical*. The ABG, sodium, potassium, calcium, magnesium, phosphate, chloride, albumin, lactate, hemoglobin, hematocrit, leukocyte, blood urea nitrogen, and creatinine values determined during the first 24-hour period were used for calculating the Acute Physiologic Assessment and Chronic Health Evaluation (APACHE II), strong ion difference apparent (SIDa), and SID effective (SIDe) scores, which were subsequently compared between the groups.

Results

Overall, 220 (110 medical and 110 surgical) patients were included. The mean patient age was 63.56 ± 18.08 years. The mean APACHE II scores were 21.99 and 19.63 in the medical and surgical groups, respectively. Overall, 110 patients died, while 110 were referred to the regular patient floor. The mean APACHE II score of the patients who died was 28.3, and the latter group had a mean APACHE II score of 13.57. There was a significant difference between the surgical and medical patient groups regarding mean values of APACHE II, SIDa, and SIDe scores. Also, the differences were significant between the patients who died and were discharged. There was a significant difference between the patients who died and were discharged regarding the strong ion gap (SIG); however, the medical and surgical patient groups were not different concerning the SIG values.

Conclusions

We conclude that SIDa, SIDe, and SIG can be used in medical and surgical ICU patients to predict prognosis.

## Introduction

Acid-base imbalance is a frequently encountered acute clinical entity. Therefore, timely diagnosis of acid-base imbalance and its accurate management are crucial for intensive care unit teams. Maintaining most biochemical reactions in the human body requires the hydrogen ion concentration to be within the physiological range. Severe acid-base imbalance was found to be associated with arrhythmia, other morbidities, and mortality. Under normal circumstances, the hydrogen ion concentration is kept in the range of 36-40 nmoI/L and the pH is maintained in the range of 7.37-7.43 by sensitive mechanisms. Henderson-Hasselbalch (HH), base excess (BE), and anion gap (AG) calculations are inadequate in assessing the acid-base imbalance in some cases [1-4]. Although criticized, the Stewart approach can assist in diagnosing some acid-base imbalance cases, which the HH approach cannot diagnose. The Stewart approach defines and analyzes the factors independently affecting the pH in vitro. These independent variables are strong ion difference (SID), carbon dioxide (CO_2_) load, and weak acid load. One of the differences between Stewart's theory and the HH approach is that pH is affected by the electrolytes (i.e., SID change) and the changes in the serum albumin concentration. In contrast to BE and AG, the Stewart approach is helpful in the identification of acid-base problems in the presence of electrolyte imbalance and hypoalbuminemia. In this study, we implemented the Stewart approach in medical and surgical ICU patients during ABG assessments. We analyzed the effectiveness of markers such as strong ion gap (SIG), SID apparent (SIDa), and SID effective (SIDe). These markers are not routinely used to demonstrate acid-base imbalances and cannot be optimally diagnosed by the HH, AG, and BE methods.

## Materials and methods

This study was designed as a single-center retrospective study. It was approved by the Izmir Katip Celebi University Ataturk Training and Research Hospital Ethical Review Committee for Noninterventional Studies. Data of the adult (i.e., age > 18 years) patients admitted to the anesthesiology and reanimation intensive care unit (ICU) during a one-year study period were retrospectively reviewed. Patients who were older than 18 years of age, had been hospitalized in intensive care for more than 24 hours, and whose blood samples could be taken within the time specified in the study after admission to the ICU were included in the study. Patients who stayed in the ICU for shorter than 24 hours, those who did not undergo ABG sampling, and those who were transferred to another hospital were excluded. Primary diagnosis, age, gender, the reason for ICU referral, and comorbidities of all patients were recorded in an electronic database. Data of the postoperative (i.e., surgical) patients were recorded separately; the indication for surgery was also included. The Acute Physiologic Assessment and Chronic Health Evaluation (APACHE II) score was calculated, and the laboratory parameters of all patients were recorded in the 30th minutes of admission to the ICU [5].

The SIDa, SIDe, and SIG values were calculated using the following formulas through the webpage http://www.acidbase.org [5,6]. ATOT was used as the abbreviation of total plasma concentration of inorganic phosphate, serum proteins, and albumin (weak nonvolatile acids):

$ATOT=(Alb)x(0.123pH&minus;0.631)+(Pi)x(0.309pH&minus;0.469)$

$SIDa=(Na+ +K+ +Mg2++Ca2+)&minus;(Cl&minus;+ lactate+urate)$

$SIDe= [ Weak acids-] +[HCO3]$

$SIG=SIDa&minus;SIDe$

While the SIDa values were categorized as low (<40 mEq/L), normal (40-46 mEq/L), and high (>46 mEq/L), the SIDe values were classified as low (<36 mEq/L), normal (36-40 mEq/L), and high (>40 mEq/L).

Comparisons were made between the patients who died in the ICU and those who were discharged from the ICU regarding the gender distribution, age, treatment regimens, APACHE II, SIDa, SIDe, and SIG values in the 30 days. A similar comparison was made between the *medical* and *surgical* patients.

Statistical analysis

All statistical analyses were performed using IBM Corp. Released in 2010. IBM SPSS Statistics for Windows, Version 19.0 (IBM Corp., Armonk, NY). The Mann-Whitney U-test was used to distribute of the patients based on age and gender. Independent samples test and Mann Whitney U-tests were performed to analyze the age, APACHE II score, SIDa, SIDe, and SIG values of the surgical and medical patients. For comparative analysis of age, APACHE II, SIDa, SIDe, and SIG values of the surgical and medical patients based on the status at the time of discharge, the Mann-Whitney U-test was used.

## Results

Overall, 220 (110 medical and 110 surgical) patients were included. The mean patient age was 63.56 ± 18.08 years. While 39.1% (*n *= 86) of these patients were female, 60.9% (*n *= 134) were male. There was no significant difference between the female and male patients regarding the mean patient age (*P* > 0.05) (Table 1).

**Table 1:** Distribution of the patients based on age and gender. SD, standard deviation

			Age (years)		*P*-value
	n	%	Mean ± SD	Min.-Max.	
Gender					
Female	86	39.1	63.5 ± 19.71	19-93	0.745
Male	134	60.9	63.6 ± 17.02	19-91	
Total	220	100	63.56 ± 18.08	19-93	

The distribution of diagnoses of cases according to their treatment method is indicated in Table 2.

**Table 2:** Distribution of diagnoses of cases according to their treatment methods. ERCP, endoscopic retrograde cholangiopancreatography; COPD, chronic obstructive pulmonary disease

	Surgical/Medical				Total	
	Surgical		Medical			
	n	%	n	%	n	%
Acute abdomen	16	14.5	-	-	16	7.3
Acute cholecystitis	-	-	1	0.9	1	0.5
Acute subdural hematoma	2	1.8	-	-	2	0.9
Appendicitis	1	0.9	-	-	1	0.5
Nonvehicular trauma	5	4.5	1	0.9	6	2.7
Neck dissection	1	0.9	-	-	1	0.5
Multiple organ dysfunction	-	-	2	1.8	2	0.9
Diabetic foot ulcer	1	0.9	-	-	1	0.5
Diabetic ketoacidosis	-	-	1	0.9	1	0.5
Duodenal ulcer perforation	6	5.5	-	-	6	2.7
Falls injury	2	1.8	1	0.9	3	1.4
Eclampsia	1	0.9	-	-	1	0.5
Perforation in ERCP	1	0.9	-	-	1	0.5
Sepsis after ERCP	1	0.9	1	0.9	2	0.9
Evisceration	1	0.9	-	-	1	0.5
Femur fracture	1	0.9	-	-	1	0.5
Fournier gangrene	3	2.7	-	-	3	1.4
Fulminant hepatitis	-	-	1	0.9	1	0.5
General condition disorder	-	-	11	10	11	5
Gastrointestinal system hemorrhage	-	-	1	0.9	1	0.5
Hypertensive pulmonary edema	-	-	2	1.8	2	0.9
Hypertensive cerebellar hemorrhage	-	-	1	0.9	1	0.5
Ileus	12	10.9	-	-	12	5.5
Immune deficiency	-	-	1	0.9	1	0.5
Incarcerated hernia	1	0.9	-	-	1	0.5
Intoxication	-	-	2	1.8	2	0.9
Intraabdominal sepsis	-	-	2	1.8	2	0.9
Open feeding jejunostomy	1	0.9	-	-	1	0.5
Intracranial mass	1	0.9	-	-	1	0.5
COPD	-	-	8	7.3	8	3.6
COPD with pneumonia	-	-	1	0.9	1	0.5
Choledochus perforation	1	0.9	-	-	1	0.5
Colonic perforation	1	0.9	-	-	1	0.5
Lumbar abscess	1	0.9	-	-	1	0.5
Lumbar fracture	3	2.7	-	-	3	1.4
Meningitis	-	-	1	0.9	1	0.5
Mesenteric ischemia	5	4.5	-	-	5	2.3
Pregnancy with intrauterine fetal death	1	0.9	-	-	1	0.5
Multiple trauma	5	4.5	4	3.6	9	4.1
Necrotizing pancreatitis	1	0.9	-	-	1	0.5
Postoperative glioblastoma multiforme	-	-	1	0.9	1	0.5
Pancreas cancer	4	3.6	-	-	4	1.8
Pancreatitis	-	-	1	0.9	1	0.5
Parathyroid adenoma-hypercalcemia	-	-	1	0.9	1	0.5
Perforation	14	12.7	-	-	14	6.4
Peritonitis	1	0.9	-	-	1	0.5
Pneumonia	-	-	33	30	33	15
After cardiopulmonary resuscitation	-	-	15	13.6	15	6.8
Pre-eclampsia	4	3.6	-	-	4	1.8
Pulmonary embolism	-	-	3	2.7	3	1.4
Retroperitoneal abscess	1	0.9	-	-	1	0.5
Sepsis	-	-	6	5.5	6	2.7
Sepsis (After ERCP)	-	-	1	0.9	1	0.5
Sepsis ( Pancreatic cyst)	-	-	1	0.9	1	0.5
Septic arthritis	1	0.9	-	-	1	0.5
Ventriculoperitoneal shunt insertion	1	0.9	-	-	1	0.5
Akut respiratory distress	-	-	1	0.9	1	0.5
Status epilepticus	-	-	1	0.9	1	0.5
Strangulated hernia	2	1.8	-	-	2	0.9
Subarachnoid hemorrhage	1	0.9	1	0.9	2	0.9
Trauma	3	2.7	-	-	3	1.4
Urosepsis	-	-	3	2.7	3	1.4
Volvulus	1	0.9	-	-	1	0.5
Fall from height	2	1.8	-	-	2	0.9

The majority (*n *= 75, 68.2%) of the *surgical* patients were general surgery patients, while pneumonia was the most (*n *= 34, 15.45%) frequent diagnosis among the *medical* patients. The mean APACHE II scores were 21.99 and 19.63 in the medical and surgical patient groups, respectively. There were statistically significant differences between the surgical and medical patient groups concerning APACHE II scores, SIDa, and SIDe values (*P *< 0.05) (Table 3).

**Table 3:** Age, APACHE II, SIDa, SIDe, and SIG values of the surgical and medical patients. SD, standard deviation; SIG, strong ion gap; SIDa, strong ion difference apparent; SIDe, strong ion difference effective; APACHE, Acute Physiologic Assessment and Chronic Health Evaluation

	Surgical		Medical		*P*-value
	Mean ± SD	Min-Max	Mean ± SD	Min-Max	
Age (years)	61.32 ± 17.34	19-90	65.8 ± 18.59	19-93	0.024
APACHE II	19.63 ± 9.03	5-42	21.99 ± 7.39	3-39	0.035
SIDa	41.77 ± 6.47	20.8-64.2	44.41 ± 5.62	31.13-59.9	0.001
SIDe	28.28 ± 6.09	13-45.5	30.43 ± 7.63	13-53.4	0.022
SIG	13.49 ± 7.3	-3.5 to 36.5	13.98 ± 5.79	0.8-31.31	0.160

However, this comparison did not reveal a significant difference between the two patient groups regarding SIG values (*P *> 0.05).

The analysis of the patients discharged from the ICU showed that the mean SIDa, SIDe, and SIG values were, respectively, 41.2, 29.78, and 11.43 in the surgical and 46.05, 33.01, and 13.04 in the medical patient groups. While 110 patients died, 110 were discharged from the ICU. Comparison of these two patient groups in terms of APACHE II scores and SIDa, SIDe, and SIG values revealed statistically significant differences regarding the APACHE II, SIDa, and SIDe (*P *< 0.05). However, there was no significant difference between the groups concerning the other variables (*P *> 0.05) (Table 4).

**Table 4:** Age, APACHE II, SIDa, SIDe, and SIG values of the patients based on the status at the time of discharge. SD, standard deviation; SIG, strong ion gap; SIDa, strong ion difference apparent; SIDe, strong ion difference effective; APACHE, Acute Physiologic Assessment and Chronic Health Evaluation

		Surgical		Medical		p value
		Mean±SD	Min-Max	Mean±SD	Min-Max	
Exitus	AGE	67.4±14.37	24-89	68.77±15.96	20-93	0.467
	APACHE II	28.38±5.74	19-42	25.2±6.26	9-39	0.017
	SIDa	42.6±7.27	27.8-64.2	43.28±5.47	31.13-56.2	0.419
	SIDe	26.13±7.17	13-45.5	28.64±7.58	14.79-53.4	0.113
	SIG	16.47±8.66	4.4-36.5	14.64±6.43	0.8-31.31	0.549
Discharge	AGE	57.11±18.05	19-90	61.51±21.31	19-93	0.150
	APACHE II	13.57±5.02	5-25	17.36±6.41	3-28	0.001
	SIDa	41.2±5.84	20.8-52.1	46.05±5.49	31.2-59.9	0.000
	SIDe	29.78±4.73	19-42.9	33.01±7	13-51.3	0.005
	SIG	11.43±5.35	-3.5-23.7	13.04±4.62	2.3-21.6	0.083

In the surgical patient group, significant differences were observed between the discharged and deceased patients in terms of APACHE II, SIDe, and SIG values (*P* < 0.05). There were significant differences between medical ICU patients who were discharged and those who died, with respect to APACHE II, SIDa, and SIDe values (*P* < 0.05). Furthermore, when comparing all patients who died with those who were discharged, significant differences were observed in APACHE II, SIDe, and SIG values. The two groups were similar regarding other variables (*P *>0.05) (Table 5).

**Table 5:** Comparative analysis of the age, APACHE II, SIDa, SIDe, and SIG values of the surgical and medical patients based on the status at the time of discharge. SD, standard deviation; SIG, strong ion gap; SIDa, strong ion difference apparent; SIDe, strong ion difference effective; APACHE, Acute Physiologic Assessment and Chronic Health Evaluation

		Outcome				*P*-value
		Exitus		Discharge		
		Mean ± SD	Min-Max	Mean ± SD	Min-Max	
Surgical	Age (years)	67.4 ± 14.37	24-89	57.11 ± 18.05	19-90	0.002
	APACHE II	28.38 ± 5.74	19-42	13.57 ± 5.02	5-25	0.000
	SIDa	42.6 ± 7.27	27.8-64.2	41.2 ± 5.84	20.8-52.1	0.367
	SIDe	26.13 ± 7.17	13-45.5	29.78 ± 4.73	19-42.9	0.002
	SIG	16.47 ± 8.66	4.4-36.5	11.43 ± 5.35	-3.5 to 23.7	0.005
Medical	Age (years)	68.77 ± 15.96	20-93	61.51 ± 21.31	19-93	0.100
	APACHE II	25.2 ± 6.26	9-39	17.36 ± 6.41	3-28	0.000
	SIDa	43.28 ± 5.47	31.13-56.2	46.05 ± 5.49	31.2-59.9	0.010
	SIDe	28.64±7.58	14.79-53.4	33.01±7	13-51.3	0.001
	SIG	14.64 ± 6.43	0.8-31.31	13.04 ± 4.62	2.3-21.6	0.244
Total	Age (years)	68.21 ± 15.28	20-93	58.91 ± 19.48	19-93	0.000
	APACHE II	26.5 ± 6.23	9-42	15.12 ± 5.9	3-28	0.000
	SIDa	43 ± 6.25	27.8-64.2	43.18 ± 6.16	20.8-59.9	0.618
	SIDe	27.61 ± 7.48	13-53.4	31.1 ± 5.96	13-51.3	0.000
	SIG	15.39 ± 7.44	0.8-36.5	12.08 ± 5.11	-3.5 to 23.7	0.001

## Discussion

Patients with various diagnoses are admitted to the ICUs [7]. Different prognostic models were developed to predict the prognosis of these patients and manage them accordingly [7-12]. Also, these models are used in different ICUs at different time frames for both quality control and research purposes. Since the cost of ICU care is relatively high and the patients and their families are under challenging emotional conditions, the prediction of the prognosis of ICU patients has become a popular topic, especially during the last two decades. Of note, these patients may have very complicated acid-base imbalances due to the broad spectrum of the patients admitted to these units.

In our study, we analyzed some potential parameters to predict the prognosis of ICU patients and investigated the association of these parameters with the prognosis of these patients. We analyzed the SIDa, SIDe, and SIG values not assessed in the context of routine arterial blood gas analysis.

Fidkowski and Helstrom noted that in diagnosing patients with metabolic acidosis in critical patients, AG and SIG could have a significant value by revealing the metabolic acidosis stemming from the unmeasurable anions [13]. Story et al. noted that AG and BE could be affected by the plasma albumin level, and therefore, SIG was preferable [14]. Aligned with Story et al., we propose that the Stewart method could prove beneficial in cases of acid-base imbalance that conventional approaches cannot delineate. Ratanarat et al. stated that the Stewart method was more reliable than the traditional approach; however, it was too difficult to perform due to its complicated formulas [15].

In our study, we determined that the Stewart approach was advantageous in predicting the prognosis of ICU patients. We found that the SIG level was relatively higher in critically ill patients. Ho et al. compared the prognostic significance of SIG and the other acid-base parameters and concluded that lactate was superior to SIG [16].

As noted in the Methods section, lactate level is a parameter used while calculating the SID value. Although lactate level can be readily measured and does not necessitate a unique calculation formula, we suggest that it is not an ideal parameter for use in complex acid-base imbalance cases implicating several other variables.

Our study is the first to compare the SIG, SIDe, and SIDa subgroup values and the prognosis between the medical and surgical ICU patient groups. In this study, we calculated the SIDa, SIDe, and SIG values separately and analyzed their significance. Our results indicated a significant difference between the surgical and medical patient groups regarding age, APACHE II, SIDa, and SIDe values.

While there was a significant difference between the patients who died and were discharged concerning the SIG levels, there was no significant difference between the medical and surgical patients who died.

In the surgical and medical patient groups, comparisons regarding SIDe and SIDa revealed significant differences. Therefore we suggest that the calculations of SIDa and SIDe are more straightforward than SIG. This makes these values more easily applicable and can be used as individual indicators.

## Conclusions

In conclusion, we found that the SIG values were relatively high in medical or surgical ICU patients. However, the SIG values at admission could not serve as prognostic indicators. We observed an elevation in SIG levels among critically ill patients admitted to the ICU, and this increase correlated with clinical severity and mortality.

We think that using the Stewart approach may be useful in acid-base disorders that cannot be explained by the traditional approach. These are useful parameters that contribute to the identification, clarification, and understanding of complex metabolic acidoses caused by unmeasurable anions. The SIG values can be useful as an adjunct to AG, corrected AG, and BE for further analysis of the acid-base imbalance and patient management. Also, the changes in the SIDa and SIDe levels should be considered while evaluating the SIG levels. However, studies investigating other mortality-related biomarkers and SIG are needed to delineate SIG's clinical role further.

## References

[1] Matoušovic K, Havlín J, Schück O (2016). Clinical evaluation of acid-base status: Henderson-Hasselbalch, or Stewart-Fencl approach?. Cas Lek Cesk.

[2] Magder S, Emami A (2015). Practical approach to physical-chemical acid-base management. Stewart at the bedside. Ann Am Thorac Soc.

[3] Story DA (2016). Stewart acid-base: a simplified bedside approach. Anesth Analg.

[4] Cove M, Kellum JA (2020). The end of the bicarbonate era? A therapeutic application of the Stewart approach. Am J Respir Crit Care Med.

[5] Elbers PW, Van Regenmortel N, Gatz R (2015). Over ten thousand cases and counting: acidbase.org is serving the critical care community. Anaesthesiol Intensive Ther.

[6] Alshiakh SM (2023). Role of serum lactate as prognostic marker of mortality among emergency department patients with multiple conditions: a systematic review. SAGE Open Med.

[7] Pronovost PJ, Nolan T, Zeger S, Miller M, Rubin H (2004). How can clinicians measure safety and quality in acute care?. Lancet.

[8] Tontu F, Aşar S, Bilgin Ören Z (2023). Stewart’s approach for acid-base disorders: does the strong ion difference and effects have an impact on intensive care unit mortality?. Turkish Journal of Intensive Care.

[9] Schork A, Moll K, Haap M, Riessen R, Wagner R (2021). Course of lactate, pH and base excess for prediction of mortality in medical intensive care patients. PLoS One.

[10] de Meneses F, Bezerra I, Ribeiro E, Furtado A Junior, Peixoto A Junior (2007). Base excess and early mortality in patients admitted to the general intensive care unit at a university hospital in Fortaleza. Crit Care.

[11] Berndtson AE, Palmieri TL, Greenhalgh DG, Sen S (2013). Strong ion difference and gap predict outcomes after adult burn injury. J Trauma Acute Care Surg.

[12] Haas SA, Lange T, Saugel B, Petzoldt M, Fuhrmann V, Metschke M, Kluge S (2016). Severe hyperlactatemia, lactate clearance and mortality in unselected critically ill patients. Intensive Care Med.

[13] Fidkowski C, Helstrom J (2009). Diagnosing metabolic acidosis in the critically ill: bridging the anion gap, Stewart, and base excess methods. Can J Anaesth.

[14] Story DA, Poustie S, Bellomo R (2002). Estimating unmeasured anions in critically ill patients: anion-gap, base-deficit, and strong-ion-gap. Anaesthesia.

[15] Ratanarat R, Sodapak C, Poompichet A, Toomthong P (2013). Use of different approaches of acid-base derangement to predict mortality in critically ill patients. J Med Assoc Thai.

[16] Ho KM, Lan NS, Williams TA, Harahsheh Y, Chapman AR, Dobb GJ, Magder S (2016). A comparison of prognostic significance of strong ion gap (SIG) with other acid-base markers in the critically ill: a cohort study. J Intensive Care.

